# Relationships between the magnitude of representational momentum and the spatial and temporal anticipatory judgments of opponent’s kicks in taekwondo

**DOI:** 10.3389/fpsyg.2023.1193116

**Published:** 2023-09-21

**Authors:** Kuniyasu Imanaka, Takahiro Sugi, Hiroki Nakamoto

**Affiliations:** ^1^Department of Health Promotion Sciences, Tokyo Metropolitan University, Tokyo, Japan; ^2^Graduate School of Humanities [Psychology], Tokyo Metropolitan University, Tokyo, Japan; ^3^Faculty of Physical Education, National Institute of Fitness and Sports in Kanoya, Kagoshima, Japan

**Keywords:** representational momentum, coincidence timing, kick-type judgment, anticipation, taekwondo kick, sport expertise

## Abstract

For successful actions in a fast, dynamic environment such as sports, a quick successful anticipation of a forthcoming environmental state is essential. However, the perceptual mechanisms involved in successful anticipation are not fully understood. This study examined the relationships between the magnitude of representational momentum (RM) as a forward displacement of the memory representation of the final position of a moving object (which implies that observers perceptually “see” a near future forthcoming dynamic environmental state) and the temporal and spatial anticipatory judgments of the opponent’s high or middle kicks in taekwondo. Twenty-seven participants (university taekwondo club members and non-members) observed video clips of taekwondo kicks that vanished at one of 10 frame positions prior to the kick impact and performed three tasks consecutively: anticipatory coincidence timing (CT) with the arrival of kick impact, judgment of the kick type (high and middle kicks) by forced choice, and judgment of the vanishing frame position (measuring RM). Our results showed significant group effects for the number of correct kick-type judgments and the judgment threshold for kick-type choice (kick-typeJT), which was estimated in terms of individual psychometric function curves. A significant correlation was found between the magnitude of RM (estimated at kick-typeJT) and kick-typeJT, but not between the CT errors (estimated at kick-typeJT) and kick-typeJT. This indicates that the magnitude of RM may play an influential role in quick kick-type judgments, but not in coincidence timing while observing an opponent’s kick motion. These findings suggest that subjective anticipatory perception or judgment of the future spatial state is vital to anticipatory actions under severe time constraints.

## Introduction

Representational momentum (RM) was first hypothesized by Freyd (1983) and Freyd and Finke (1984) as a forward displacement of the memory representation of the final position of a moving stimulus. Hubbard and Bharucha (1988) further examined RM using the linear movement of a dot stimulus that suddenly disappeared, asking participants to judge the vanishing point of the moving dot. The results showed that the judged vanishing point was displaced forward in the direction of the path of the stimulus movement rather than at the actual vanishing point. This implies that observers tend to perceptually “see” the vanishing point of a moving object at a future location that the moving object would reach. Thus far, many studies have comprehensively examined the nature of RM, providing evidence for various aspects of its perceptual and cognitive characteristics such as stimulus velocity (Finke et al., 1986; Getzmann and Lewald, 2009; Feinkohl et al., 2014; Merz et al., 2019), eye movements (Kerzel, 2000), attention (Hayes and Freyd, 2002; Kerzel, 2003; Hubbard et al., 2009), development (Hubbard et al., 1999; Perry et al., 2008; Shirai et al., 2018; Mori et al., 2022), and aging aspects (Piotrowski and Jakobson, 2011). Previous studies on RM generally provided evidence for forward displacement in the perceptual or memory representations of a moving object (see Hubbard, 2005, 2014, 2015, for reviews).

The salient, fundamental nature of RM suggests that it may compensate for neural processing delays (Kerzel and Gegenfurtner, 2003; Nijhawan and Kirschfeld, 2003; see Hubbard, 2005 for a review). Neural processing delays occur in neural processes from stimulus inputs to the resultant perception of sensory inputs, with delays elapsing at least tens of milliseconds in visual processing (Schmolesky et al., 1998). This implies that when a memory or perceptual representation of a moving object arises in the brain, the moving object in the environment would have already moved further to a future location during processing. Unless RM occurs, neural processing delays could result in a spatial (and temporal) gap between the actual environmental features and perceptual representational ones of the moving object. This indicates that RM may compensate for neural processing delays, thus yielding temporal consistency between the dynamic events occurring in the environment and their neural representation in the brain.

Perceptual or cognitive consistency between a dynamic environmental event and its neural representation in the brain may be essential for dynamic perceptual-motor processes (e.g., prediction, expectation, and motor preparation and execution). This is most typical in sports performances such as catching or hitting a fast-moving ball, offending or defending against opponent players in ball games, and martial arts, with probable accurate coincidence timing (CT), which is based on the judgment of time-to-contact (TTC), with those of dynamic environmental events. Therefore, the RM may be beneficial for successful perceptual and motor performance in sports and other dynamic activities. Several studies have provided empirical evidence that experts in various behavioral domains such as those in basketball (Gorman et al., 2011, 2012), baseball (Nakamoto et al., 2015), badminton (Jin et al., 2017), rugby (Anderson et al., 2019), dance (Chen et al., 2019), aircraft pilots (Blättler et al., 2011), and automobile drivers (Thornton and Hayes, 2004; Blättler et al., 2010, 2012) exhibit larger magnitudes of RM than do novices or non-experts when using stimuli representing actual domain-specific situations. The large magnitude of RM among experts is assumed to develop through long-term experiences of seeing and responding to moving objects during specific perceptual-motor activities. In general, RM indicates that observers can “see” a moving object at a displaced location as the actual real-time location in the real world. Therefore, RM could benefit observers in anticipating the future scene of a moving object in many sports (e.g., basketball, baseball, badminton, rugby, and dance) and other dynamic perceptual-motor activities (e.g., landing an aircraft and driving a car).

We (Nakamoto et al., 2015) previously examined the relationship between the magnitude of RM and temporal anticipation using a CT task among baseball players and non-players. In our study, participants performed the CT task and a task for measuring the magnitude of RM. In the experiment, a moving stimulus with two velocity conditions, fast (15 m/s, duration = 0.27 s) and slow (10 m/s, duration = 0.4 s), ran on a 4 m linear track, along which 200 light-emitting diodes (LEDs) were set at every 2 cm. Through successive light on/off cycles of the 200 LEDs, an apparent movement was made on the track. In half the trials, the moving stimulus was visually occluded from the middle to the end of the linear track. In the other half, the moving stimulus reached the end of the linear track without any visual occlusion. Participants performed the CT task by pressing a response key with the anticipatory judgment of the arrival of the moving stimulus at the end of the linear track, irrespective of the two conditions, with and without visual occlusion. Participants then performed a task for measuring the magnitude of the RM, in which they verbally reported the perceived last position (i.e., subjective vanishing position) of the moving stimulus by choosing a numbered label set in line with the LEDs along the linear track for both velocity conditions, with and without visual occlusion. Our results showed that in the visual occlusion condition, baseball players demonstrated significantly smaller CT errors and larger magnitudes of RM than did the non-players. Our results also showed significant negative correlations between the CT error and RM magnitude for both baseball players and non-players in the fast velocity condition, but this appeared only for the baseball players in the slow velocity condition. This finding is consistent with those in Gray and Thornton’s (2001) study on TTC (which is associated with CT) and RM. They reported that the magnitude of RM was negatively correlated with TTC errors under a long stimulus duration (>1 s) or a slow velocity condition for non-expert participants, which was not the case when RM was absent. The findings of Gray and Thornton (2001) and Nakamoto et al. (2015) further suggest that RM may contribute to anticipatory judgments for moving stimuli in the CT and TTC task conditions. In the sports domain, many studies reported that the magnitude of RM is larger for experts than for novices when using domain-specific stimuli (Gorman et al., 2011, 2012; Nakamoto et al., 2015; Jin et al., 2017; Anderson et al., 2019; Chen et al., 2019). However, the issue of whether the large RM of experts with the use of domain-specific stimuli immediately benefits their excellent anticipatory judgments in sports has not yet been fully examined. A few studies such as that by Nakamoto et al. (2015) provide evidence for the relationship between the magnitude of RM and accuracy of CT in sports-like situations.

The present study examined whether the magnitude of RM correlates with the temporal and spatial anticipatory judgments of taekwondo players’ offensive kicks. Combat sports such as taekwondo and karate are among the fastest sports in which players are expected to respond to an opponent’s attack under severe time constraints. For instance, in taekwondo, the time taken by an opponent to strike a defender’s body is comparable to or less than the time required to react (Falco et al., 2009; Sant’Ana et al., 2016; Martínez and Bennett, 2019). Specifically, the only way to avoid an opponent’s attack is to anticipate the spatial location and temporal timing of the kick before its arrival. Research on expertise in combat sports has proposed that action anticipation, that is, the anticipatory judgment of an opponent’s movement that is likely to occur as a forthcoming action outcome, is a critical skill (Martínez and Bennett, 2019). Indeed, skilled combat players can estimate future outcomes more quickly than less-skilled players using information about early body movements from observed attacks (Williams and Elliott, 1999; Mori et al., 2002; Hagemann et al., 2010; Rosalie and Müller, 2013; Shin and Lin, 2016). However, the exact mechanisms that facilitate action anticipation and the mechanisms by which skilled players gain an enhanced ability to predict opponents’ actions more quickly and accurately remain unclear. Many studies on RM have provided evidence that forward displacement occurs in perception or judgment of the final (vanishing) position of a moving object or of a human action (Freyd, 1983; Senior et al., 2000; Verfaillie and Daems, 2002; Thornton and Hayes, 2004; Wilson et al., 2010; Hudson et al., 2016a,b), especially in sports (Anderson et al., 2019; Chen et al., 2019). Therefore, RM may be a potential mechanism for anticipatory judgment and perceptual-motor performance in skilled combat players.

The present study examined the relationship between the magnitude of RM and spatial and temporal judgments of the opponent’s kick in taekwondo by testing participants recruited from the members of a university taekwondo club and other university students (non-members). A video clip (120 frames/s) of an offensive player’s high- or middle-turning kick was presented on a monitor screen facing participants as an experimental visual stimulus. The visual stimulus then vanished at a certain frame (one of the 54 to 0 frame positions) prior to the kick impact frame of the clip. After watching the video clip suddenly vanished, participants consecutively performed three tasks: (1) CT with the estimated arrival of the kick impact; (2) judgment of the kick type (high or middle kick); and (3) judgment of the vanishing frame position, which was used to calculate the magnitude of RM as the gap in frame positions between the actual and perceived/judged vanishing frame positions. The CT task was used to measure the accuracy of CT or TTC with the estimated arrival of the kick impact at various vanishing frame positions of kick motion. Accurate CT (or TTC) performance should help in defending against an opponent’s attack by the kick at an accurate arrival time. For the kick-type judgment task, the number of correct kick-type judgments was measured at various vanishing frame positions. Accurate and quick/early judgments of kick type should help in defending against an opponent’s kick that comes at either the high or middle level of the observer’s body. For these reasons, we used the CT task and kick-type judgment task in relation to the magnitude of RM, which we measured using kick-motion video clips as domain-specific stimuli.

The following three variables were compared between club members and non-members: temporal (CT) and spatial (kick type) anticipatory judgments and the magnitude of RM. The relationships among the three variables were then examined using correlation analyses to determine whether the magnitude of RM correlated with the temporal and spatial anticipatory judgments of taekwondo kicks. Our provisional predictions were that the club member group would show more accurate CT, higher correct rates for kick-type judgments, and larger magnitudes of RM than those in the non-member group, and that the magnitude of RM correlated with CT errors and/or kick-type judgments, similar to previous findings on anticipatory capacities and RM in sports experts.

## Methods

### Participants

In total, 27^1^ participants—14 members of a university taekwondo club and 13 non-members—participated in the study. Among the 27 participants, 2 club members and 1 non-member demonstrated very early CT performances, approximately 300 ms earlier than the arrival time of a kick impact. They seemed to respond to the CT with toe-off (which occurred approximately 340 ms on average before the kick impact) rather than with kick impact. Thus, these early responses were classified as outliers, resulting in 12 club members and 12 non-members being selected for subsequent analyses.

The taekwondo club members consisted of 6 males and 6 females, aged 20.2 (SD = 1.03) years, with 5.4 (SD = 5.67) years of sports experience, including 2.6 (SD = 2.06) years of taekwondo training. Five taekwondo club members were the final winners, and four other members were among the first six places in the Japan National College Competition games. This indicated that 9 of the 12 club members were rated relatively high among university taekwondo athletes in Japan. The non-members consisted of 7 males and 5 females, aged 19.5 (SD = 1.88) years, with 5.3 (SD = 4.52) years of sports experiences, such as tennis, table tennis, baseball, football, and track and field, whose performance records were not as high as university athletes in the respective sports in Japan. Thus, “non-members” means non-taekwondo club members and does not mean novices of sports or non-athletes. No significant differences were found between the two groups concerning age (*t*_22_ = 1.076, *p* = 0.294, Cohen’s *d* = 0.439) or length of sports experience (*t*_22_ = 0.070, *p* = 0.945, Cohen’s *d* = 0.028), which included years of training in taekwondo and other sports. All participants provided their informed consent before participating in the experiments and were paid for their participation. The Ethics Committee of the Tokyo Metropolitan University approved the study. The experiments were performed according to the approved conditions and guidelines of the Declaration of Helsinki.

### Apparatus

The experiments were conducted in a soundproof darkroom to isolate the participants from visual and auditory noises other than the experimental visual stimuli. The participants sat comfortably on a chair facing a 23-inch liquid crystal display screen (Samsung, S23A700D, 1,920 × 1,080, 120 Hz refresh rate) with their chin positioned on a chin rest to maintain a viewing distance of approximately 40 cm from their eyes to the monitor screen. A personal computer system (Diginnos PC, Thirdwave Corp.), located outside the soundproof darkroom, presented the experimental visual stimuli on the monitor screen and collected participants’ responses with a software programmed by the author KI using the programming language Presentation (Neurobehavioral Systems, Inc.) run on a personal computer system. A gamepad controller (JC-U2410TB, Elecom) was used to record participants’ responses during the experimental tasks. Six push buttons of the gamepad controller were specified for participants’ judgments in each task (see the “experimental tasks” section). A button was used for the CT task. Four buttons were used for kick type judgments (high and middle kicks of the left and right legs, although data on the left–right responses were not collected for the task scores). These four buttons were also used for the RM task, where an upper or lower button (regardless of the left and right buttons) was used to choose a forward (subsequent) or backward (previous) frame for a perceived vanishing frame position. The remaining button was used to cancel the trials.

### Visual stimuli

**Figure 1:**
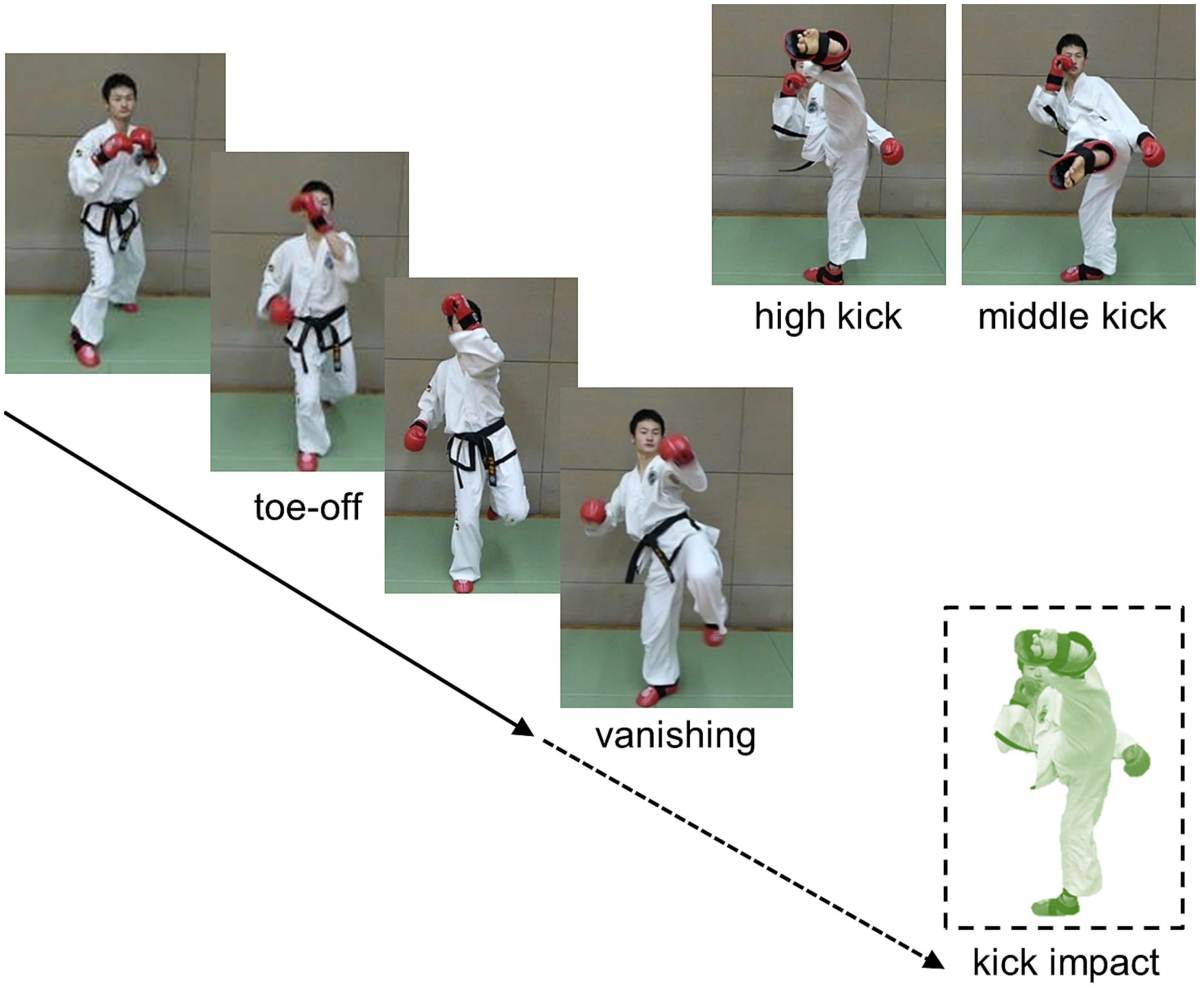
Video clip presentation of high and middle turning kicks as visual stimuli.

Computer-edited video clips comprising high- and middle-taekwondo turning kicks were used as the experimental visual stimuli (Figure 1). Four types of taekwondo turning kicks (high and middle kicks with the left and right legs) were performed with an assumed target for the kick impact 10 times each by four taekwondo expert demonstrators (other than the participants) wearing a white uniform who stood against a plain background wall of the university gymnasium. All kick demonstrations were video-recorded in complete view of the kick motion using a video camera (120 Hz sampling rate) set at the assumed target for the kick impact at a height of 1.7 m and distance of 2 m (i.e., defender’s viewpoint) from the kick demonstrator. In total, 160 video clips were recorded. Each video clip was decomposed into 120 still frames per second and used as experimental visual stimuli. The still frame pictures were sequentially presented in a 17.0 × 9.5 cm (24.4 × 13.6° in visual angle) size on the center of the monitor display screen at every frame according to a 120 Hz refresh rate. The video clips (i.e., sequential still frames) as visual stimuli suddenly blacked out (thus, the kick motion suddenly vanished) at one of the 10 frame positions set every six frames prior to the frame position of the assumed kick impact (Frame 0), resulting in 0, −6, −12, −18, …, and −54 frames set as blacking-out frame positions (labeled “vanishing frame positions” in this article). The assumed kick impact frame, or frame 0, was operationally defined as the frame in which the kick (foot) swinging motion switched from the forward to the backward swinging phase.

For each condition of the 10 vanishing frame positions, the 4 types of kicks (i.e., the high and middle kicks using the left and right legs) by the 4 demonstrators were presented twice in random order, resulting in 16 different video clips appearing twice; thus, 32 video clip presentations were presented per vanishing frame position, resulting in 320 video clips. This enabled systematic sudden vanishing while presenting video clips at 10 vanishing frame positions, including the kick impact frame. For all 320 video clips, the mean frame interval from the beginning of a vertical up- and -down motion repeatedly performed with both bare feet on the floor to the toe-off of the kicking leg or foot (defined as the frame when the toe of the kicking leg/foot left the floor to initiate the kick swinging motion) was 25.7 frames (SD = 9.49), that is, 214.5 ms (SD = 79.05). The mean frame interval from the toe-off to the assumed kick impact was 40.7 frames^2^ (SD = 3.15), that is, 339.5 ms (SD = 26.26). Several earliest vanishing frame positions at −54, −48, and −42 thus occurred at approximately 14, 8, and 2 frames (116.7, 66.7, and 16.7 ms, respectively) preceding the toe-off frame position, respectively, where the participants could see some up-down preparatory motion alone before the occurrence of the blackout of the video clip (i.e., kick motion vanishing).

### Experimental tasks

In each trial, a video clip was presented. It suddenly vanished at one of the 10 frame positions, followed by 3 tasks: (1) CT with an estimated arrival of the kick impact, (2) judgment of kick type by forced choice, and (3) estimation of the vanishing frame position.

For the CT task, participants pressed a specified button on the gamepad controller at the estimated arrival time of the kick impact. Participants could cancel^3^ their responses by pressing the cancel button on the gamepad controller when they felt they had made a mistake (pressing the button too early, late, or failing to press) and then restart the trial with a different kick video clip and different vanishing frame position.

For the kick-type judgment task, participants pressed one of four specified buttons on the gamepad controller to choose a kick (either a high or middle kick, regardless of the left and right legs). They could change their answers on type of kick until their final choice. The kick-type judgment task was performed according to the method of constant stimuli, with kick-type judgment responses performed at 10 respective vanishing frame positions presented in random order.

The estimated vanishing frame position was measured using the adjustment method, where a probe frame picture (one of the 10 frame pictures differing ±1, 2, 3, 4, and 5 frames from the actual vanishing frame position) was presented. Participants then altered it to either the previous or subsequent frame pictures by pressing one of four specified buttons on the gamepad controller (either the upper or lower button for the subsequent or previous frame, regardless of the left and right buttons) until they determined their final adjustment. The difference between the actual and estimated vanishing frame positions was subsequently calculated to determine the magnitude of RM.

### Procedures

Participants, who were tested individually, first practiced the three tasks with a video clip in at least 10 trials until they became familiar with the appropriate use of the buttons on the gamepad controller. After completing the practice trials, the participants began the trials for data collection by pressing a button. A video clip was then presented with vanishing at one of the 10 frame positions (0, −6, −12, −18, …, and −54), following which they performed the three tasks in a fixed order^4^: the CT task was performed first, followed by the kick-type judgment task and then the task of estimating the vanishing frame position. Each task was completed using participants’ self-paced button pressing to proceed with the subsequent task. They performed 32 trials per vanishing frame position for 320 trials. After every 30th trial, a break was provided, and participants resumed the next trial using a self-paced button press. After completing all the trials, participants completed a brief questionnaire about their sports experience in years, personal records in taekwondo competitions (see the “Participants” section), and their subjective reports regarding the body parts they focused on while observing the kick motion video clips.

### Data analyses

The analyses of the three dependent variables for the three tasks were conducted in four steps using IBM SPSS (ver. 28.0) and JASP (ver.0.16). First, the respective scores of the three dependent variables were calculated based on the performance data obtained for each task. For the CT task, individual CT errors were calculated per participant by subtracting the time of impact arrival in the presented video clip from the participant’s response time to CT with the estimated arrival of the kick impact they perceived. The positive value of CT error indicated a delay beyond the actual impact arrival, whereas the negative value indicated an early response before the kick impact. For the kick-type judgment task, the number of correct kick-type judgments was counted at each vanishing frame position, resulting in a range of approximately 16 (chance level) to 32 (perfect level) correct judgments. For the task of estimating vanishing frame position, the RM magnitude was calculated per trial by subtracting the actual vanishing frame position from the estimated vanishing frame position. The resulting positive value of the magnitude of RM indicates that the perceived vanishing frame position shifts forward in the direction of the kick motion compared with the actual vanishing frame position.

Second, based on the scores of the three dependent variables (CT error, number of correct kick-type judgments, and magnitude of RM), a two-way repeated-measures multivariate analysis of variance (MANOVA) was performed with two independent variables: group (club member and non-member, a between-participants factor) and vanishing frame position (−54 to 0 prior to the impact frame, a repeated-measures factor). Subsequently, additional two-way repeated-measures analyses of variance (ANOVAs) were performed on the three dependent variables with two independent variables of the group and vanishing frame position.

**Figure 2:**
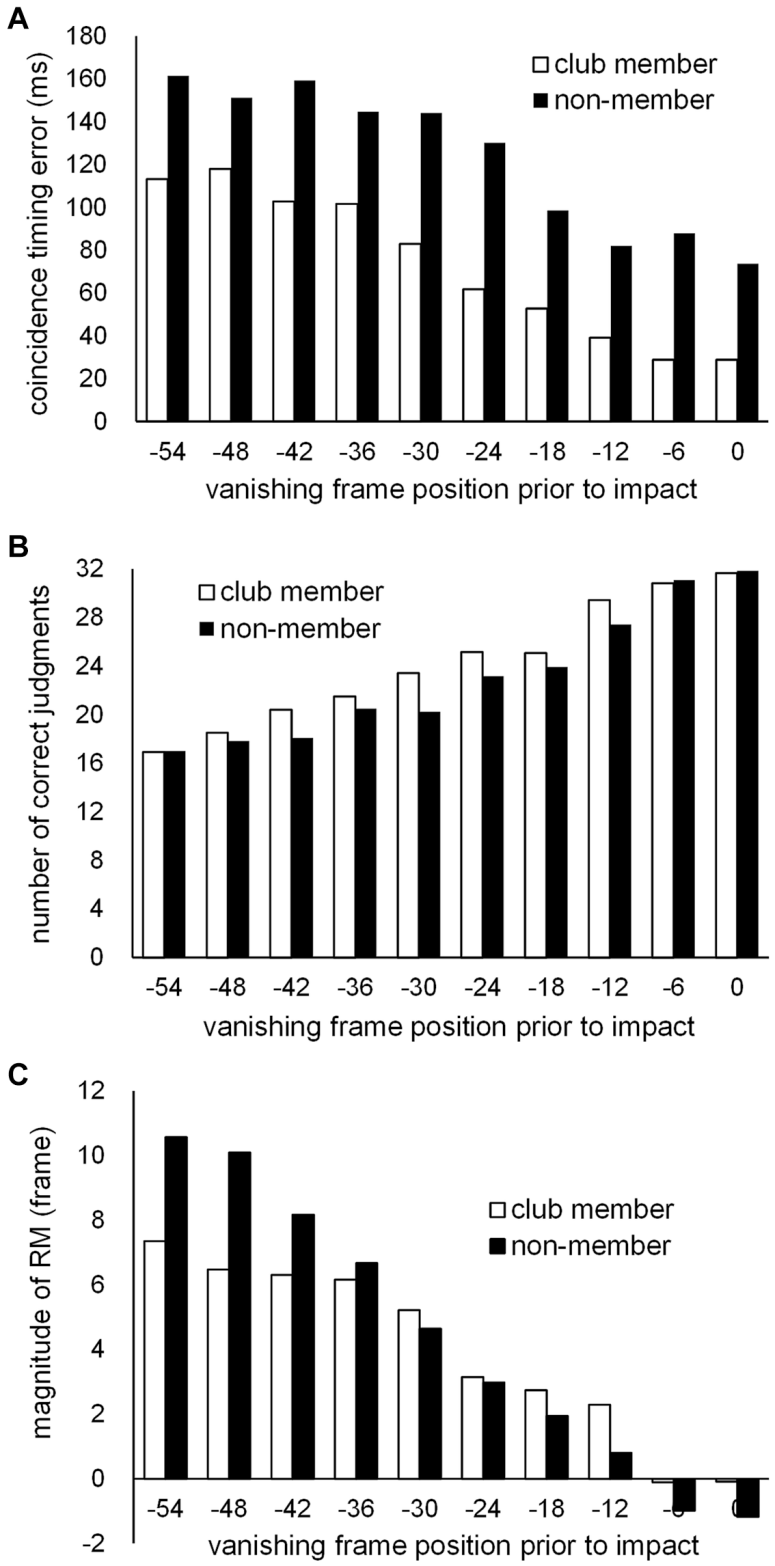
The means of **(A)** CT errors, **(B)** numbers of correct kick-type judgments, and **(C)** magnitudes of RM at 10 vanishing frame positions prior to impact frame position (Frame 0) for the club member (unfilled bar) and non-member (filled bar) groups.

Third, based on individual data regarding the number of correct kick-type judgments at each of the 10 vanishing frame positions, individual psychometric (logistic) function curves were calculated for each participant. Based on these curves, the judgment threshold for correct kick-type choice (kick-typeJT) was determined as the vanishing frame position where the correct judgment rate was 75%, namely the midpoint between the chance level (50%) and perfect level (100%) of kick-type judgments. The means of the individual kick-typeJTs of the club member and non-member groups were compared. For both CT errors and magnitudes of RM, second-degree equation curves (according to the trends in the mean scores of CT errors and magnitude of RM across the 10 vanishing frame positions; see Figures 2A–C) were calculated for each participant based on the individual data at the 10 vanishing frame positions. Subsequently, based on the individual second-degree equations, the individual CT error at kick-typeJT and magnitude of RM at kick-typeJT were estimated for each participant. The group means of the CT error estimated at kick-typeJT and magnitude of RM estimated at kick-typeJT for the club member and non-member groups were then compared.

Finally, correlation analyses were conducted to examine the potential relationships between the three variables: kick-typeJT, CT error estimated at kick-typeJT, and magnitude of RM estimated at kick-typeJT. If the correlation coefficients between any pair of dependent variables were significant, additional correlation coefficients were further examined for club member and non-member groups. Our predictions for the correlation analyses were that when the kick-typeJT appeared at an earlier phase of kick motion, the CT error (at kick-typeJT) would be smaller (more accurate), and vice versa, thus resulting in a positive correlation. Furthermore, when the magnitude of RM (at kick-typeJT) was larger (i.e., a future phase of kick motion could be perceived), kick-typeJT would appear at an earlier phase of kick motion, and vice versa, thus resulting in a negative correlation.

As a supplementary examination, we further examined the frequencies of reported words of body parts^5^ that the club member and non-member group participants focused on during the observation of the kick motion.

## Results

### Coincidence timing error, the number of correct kick-type judgments, and the magnitude of representational momentum at 10 vanishing frame positions for the club member and non-member groups

A two-way repeated-measures MANOVA was conducted on the three variables of CT error (Figure 2A), number of correct kick-type judgments (Figure 2B), and magnitude of RM (Figure 2C) for two factors (group and vanishing frame position). Our results showed that the effects of group (Wilks’ λ = 0.622, *F*_3, 20_ = 4.049, *p* = 0.021) and vanishing frame position (Wilks’ λ = 0.080, *F*_27, 573.063_ = 29.257, *p* < 0.001) were significant, as was the interaction between the group and vanishing frame position (Wilks’ λ = 0.791, *F*_27, 573.063_ = 1.772, *p* = 0.010).

Additional two-way repeated-measures ANOVAs with a Greenhouse–Geisser sphericity correction to the degree of freedom regarding the factor of the vanishing frame position were performed on the three variables. For the CT error (Figure 2A), our results showed that the main effect was not significant for the group (*F*_1, 22_ = 1.667, *p* = 0.210, *partial η*^2^ = 0.070), but was significant for vanishing frame position (*F*_1.405, 30.912_ = 9.619, *p* = 0.002, *partial η*^2^ = 0.304), with the interaction between the two factors not being significant (*F*_1.405, 30.912_ = 0.232, *p* = 0.715, *partial η*^2^ = 0.010). The overall mean CT error for all participants was 98.2 ms (SD = 96.8), showing a significant delay (*t*_23_ = 4.967, *p* < 0.001, Cohen’s *d* = 1.014) beyond the actual impact arrival. Furthermore, the mean CT error (43.6 ms, SD = 69.0) at the vanishing frame position of kick impact also showed a significant delay (*t*_23_ = 3.092, *p* = 0.005, Cohen’s *d* = 0.631). Regarding the number of correct kick-type judgments (Figure 2B), the main effects were significant for both the group (*F*_1, 22_ = 9.215, *p* = 0.006, *partial η*^2^ = 0.295) and vanishing frame position (*F*_5.170, 113.738_ = 159.601, *p* < 0.001, *partial η*^2^ = 0.879); the interaction between the two factors was not significant (*F*_5.170, 113.738_ = 1.997, *p* = 0.082, *partial η*^2^ = 0.083). For the magnitude of RM (Figure 2C), the main effect was not significant for the group (*F*_1, 22_ = 0.141, *p* = 0.711, *partial η*^2^ = 0.006), but was significant for vanishing frame position (*F*_1.722, 37.874_ = 47.571, *p* < 0.001, *partial η*^2^ = 0.684), with no significant interaction between the group and vanishing frame position (*F*_1.722, 37.874_ = 3.255, *p* = 0.056, *partial η*^2^ = 0.129). The overall mean magnitude of RM for all participants was 4.12 frames (SD = 2.76), which was significantly larger than zero (*t*_23_ = 7.310, *p* < 0.001, Cohen’s *d* = 1.492). Furthermore, the mean magnitude of RM (−0.98 frame, SD = 1.49) at the vanishing frame position of kick impact significantly differed from zero (*t*_23_ = −3.224, *p* = 0.004, Cohen’s *d* = −0.658).

### Kick-type judgment threshold, coincidence timing error and the magnitude of representational momentum (estimated at kick-type judgment threshold) for the club member and non-member groups

#### Kick-type judgment threshold

**Figure 3:**
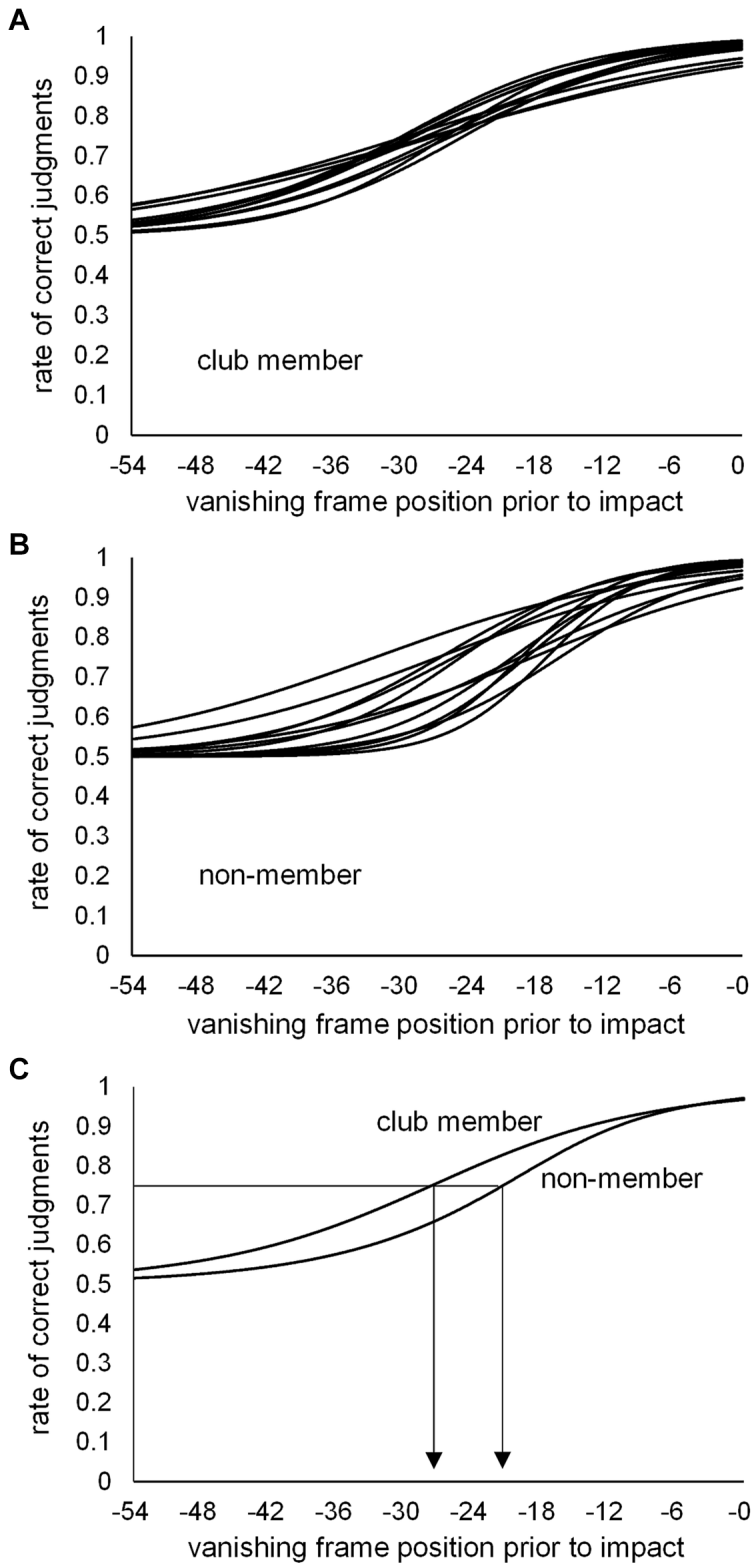
Individual psychometric function curves for **(A)** club members and **(B)** non-members, and **(C)** the mean psychometric function curves for the club member and non-member groups. The individual kick-typeJT was determined at the vanishing frame position where the correct judgment rate was 75%.

**Figure 4:**
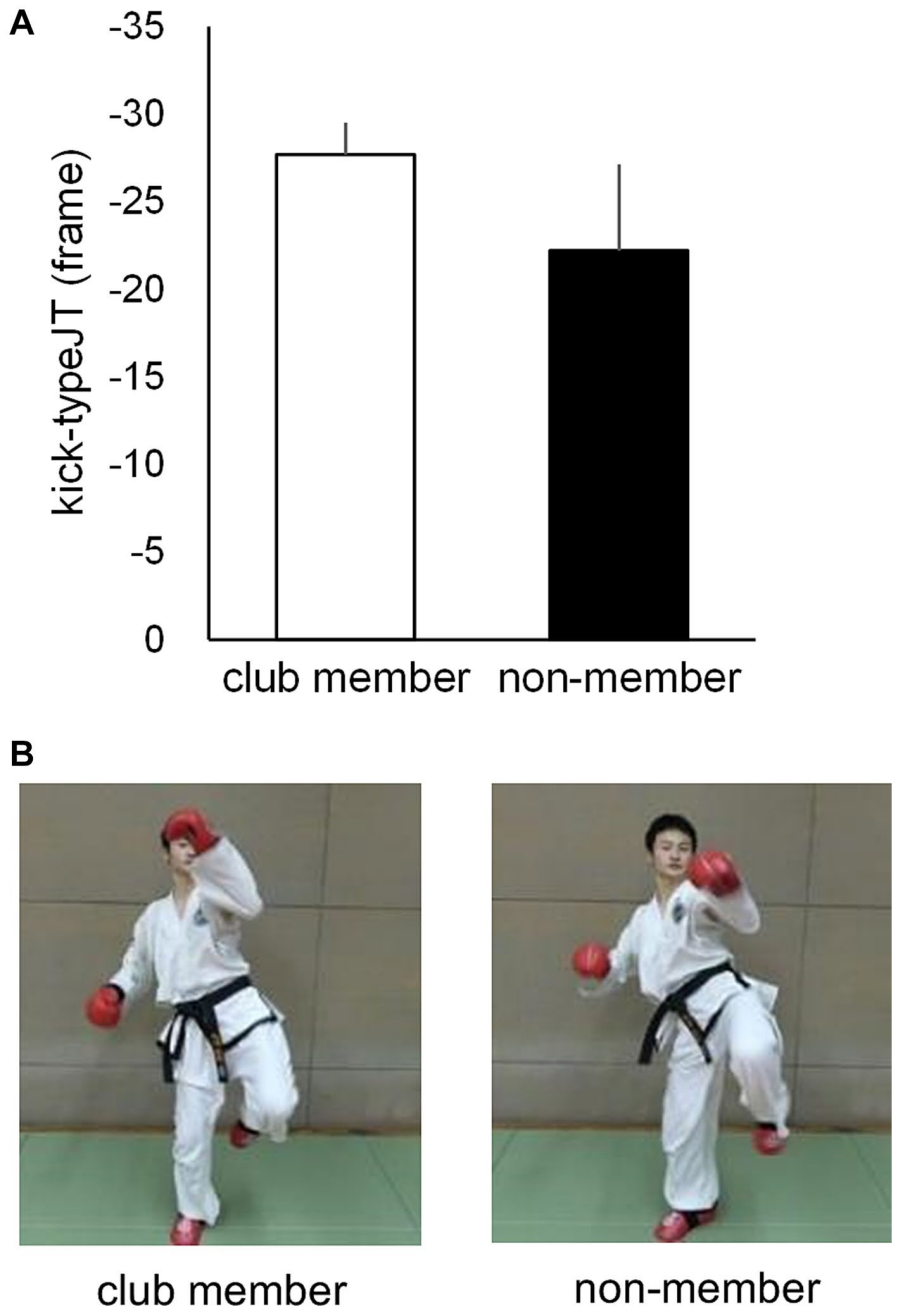
**(A)** Mean kick-typeJTs for the club member and non-member groups, with the vertical bars indicating respective SDs. **(B)** Typical kick-motion postures at the nearest frame position of mean kick-typeJTs for club member (left) and non-member (right) groups.

Based on the number of correct kick-type judgments at the 10 vanishing frame positions, individual psychometric function curves were calculated for the club member (Figure 3A) and non-member (Figure 3B) groups. Individual kick-typeJT was then calculated for each participant as the frame position, where a 75% correct judgment rate (indicating the midpoint between the perfect and chance levels of correct judgment rates) appeared on the individual psychometric function curve. The mean kick-typeJTs of the club member and non-member groups were then calculated and depicted on the mean psychometric function curves (Figure 3C). As shown in Figures 3C, 4A, the respective mean kick-typeJTs were a −27.7 (SD = 1.82) frame position (−230.8 ms) prior to the kick impact frame (Frame 0) for the club member group and −22.2 (SD = 4.93) frame position (−185.0 ms) prior to Frame 0 for the non-member group, with the group difference being significant (*t*_22_ = 3.624, *p* = 0.002, Cohen’s *d* = 1.479). The frame positions of the respective mean kick-typeJTs calculated from the mean toe-off frame position (−40.7) were 13.0 and 18.5 frame positions (108.3 and 154.2 ms) for the club member and non-member groups, respectively, indicating 31.9 and 45.5% of the mean total duration of kick motion from the toe-off to kick impact. Figure 4B shows typical kick pictures corresponding to the respective mean kick-typeJTs for the club member and non-member groups.

We further examined the difference in correct judgment rates between the club member and non-member groups at the respective group mean kick-typeJTs (see Figure 3C). At the mean kick-typeJT of the club member group (−27.7 frame position, 75% correct judgment rate), the mean correct judgment rate in the non-member group was 65.5% (SD = 8.10), which was significantly lower (*t_11_* = 4.046, *p* = 0.002, Cohen’s *d* = 1.168) than the 75% correct judgment rate. At the mean kick-typeJT of the non-member group (−22.2 frame position), the mean correct judgment rate in the club member group was 81.7% (SD = 2.54), which was significantly higher (*t_11_* = 9.187, *p* < 0.001, Cohen’s *d* = 2.652) than the 75% correct judgment rate of the non-member group.

#### Coincidence timing error estimated at kick-typeJT

**Figure 5:**
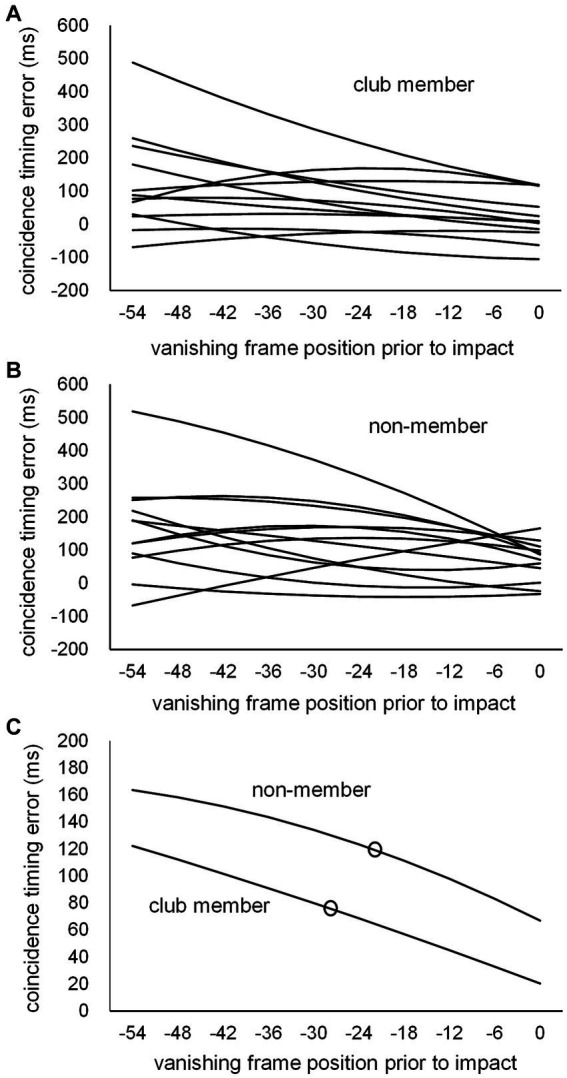
Individual second-degree equation function curves of CT errors for **(A)** club members and **(B)** non-members, and **(C)** the mean second-degree equation function curves of CT errors for the club member and non-member groups. The unfilled circles indicate those at respective kick-typeJTs in the club member and non-member groups.

**Figure 6:**
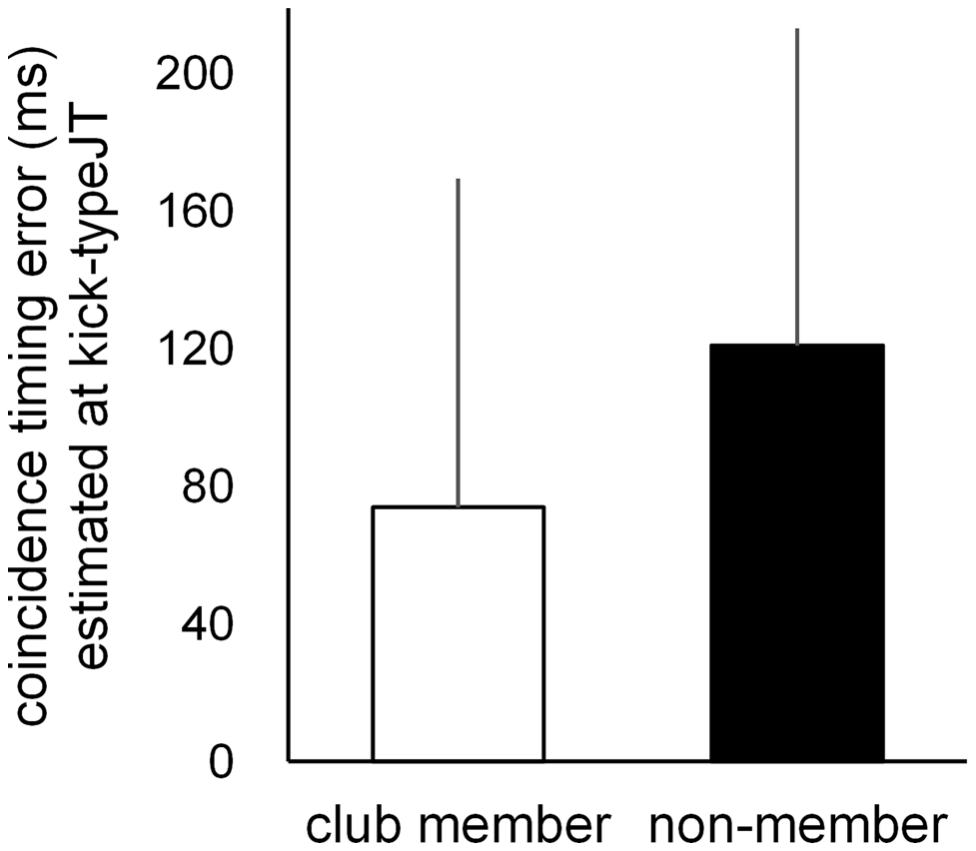
Mean CT errors estimated at kick-typeJT for the club member (unfilled bar) and non-member (filled bar) groups. The vertical bars indicate respective SDs for the club member and non-member groups.

Individual second-degree equation curves were calculated for each participant based on the CT errors at the 10 vanishing frame positions for the club member (Figure 5A) and non-member (Figure 5B) groups, with the mean second-degree equation curves for each group (Figure 5C). Based on each individual second-degree equation, individual CT errors at kick-typeJT were estimated for each participant. The mean CT errors at kick-typeJT (Figures 5C, 6) were 74.0 ms (SD = 95.44) in the club member group and 120.9 ms (SD = 92.29) in the non-member group. The mean CT error estimated at kick-typeJT in the non-member group was not significantly larger than that in the club member group (*t*_22_ = 1.225, *p* = 0.117, Cohen’s *d* = 0.500). Furthermore, as seen in Figure 5C, the mean CT error in the non-member group calculated at the club member’s kick-typeJT (−27.7 frame position) was 130.2 ms (SD = 111.70), which was not significantly larger (*t*_22_ = 1.325, *p* = 0.099, Cohen’s *d* = 0.541) than that in the club member group (74.0 ms). The mean CT error in the club member group calculated at the non-member’s kick-typeJT (−22.2 frame position) was 65.1 ms (SD = 88.19), which was not significantly smaller (*t*_22_ = 1.515, *p* = 0.072, Cohen’s *d* = 0.618) than that in the non-member group (120.9 ms).

#### Magnitude of representational momentum estimated at kick-typeJT

**Figure 7:**
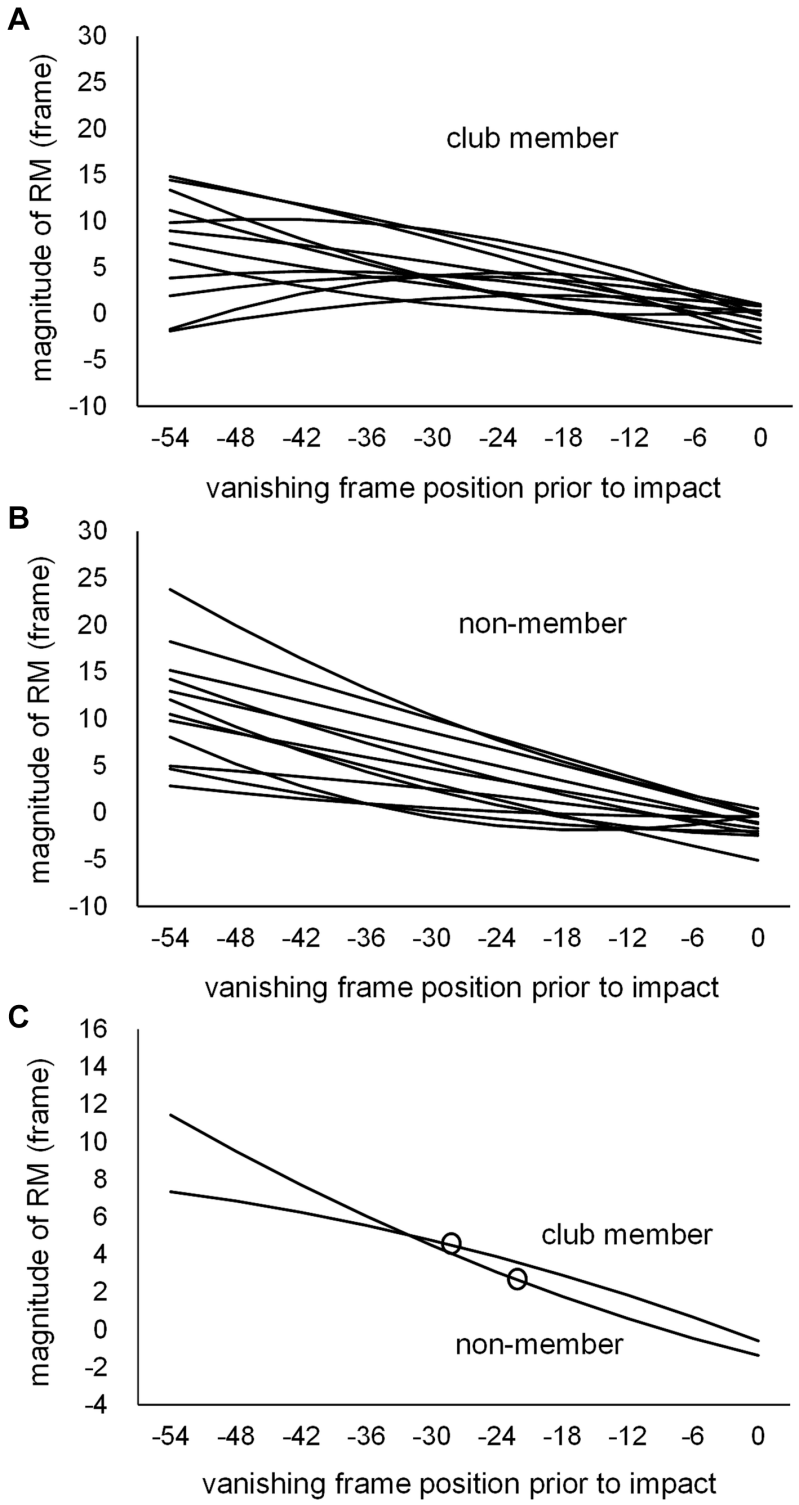
Individual second-degree equation function curves of the magnitude of RM for **(A)** club members and **(B)** non-members, and **(C)** the mean second-degree equation function curves of the magnitude of RM for the club member and non-member groups. The unfilled circles indicate those at respective kick-typeJTs in the club member and non-member groups.

**Figure 8:**
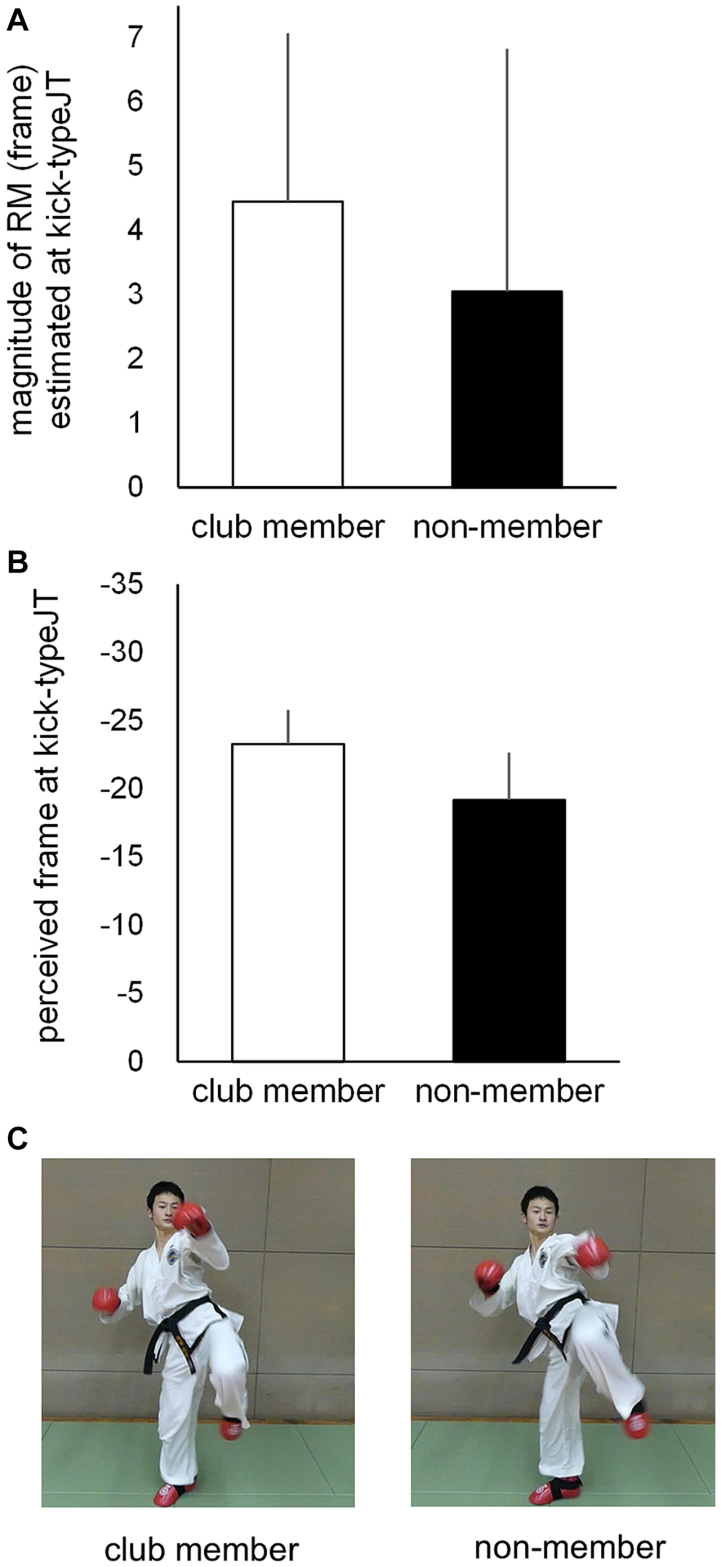
**(A)** Mean magnitudes of RM estimated at kick-typeJT and **(B)** the perceived frame positions at which kick-typeJT arose (kick-typeJT + the magnitude of RM estimated at kick-typeJT) for the club member (unfilled bar) and non-member (filled bar) groups. The vertical bars indicate respective SDs for the club member and non-member groups. **(C)** Typical kick-motion pictures at the nearest frame positions of perceived vanishing frames of kick motion for the club member (left) and non-member (right) groups.

Individual second-degree equations were calculated for each participant based on the magnitudes of RM at the 10 vanishing frame positions for the club member (Figure 7A) and non-member (Figure 7B) groups, with the mean second-degree equation curves for the respective groups (Figure 7C). The individual magnitudes of RM at kick-typeJT were then estimated per participant on each individual second-degree equation. The mean magnitudes of RM estimated at kick-typeJT were calculated, resulting in 4.4 (SD = 2.62) and 3.1 frames (SD = 3.78) for the club member and non-member groups, respectively (Figures 7C, 8A), which corresponded to 36.7 ms (SD = 21.83) and 25.8 ms (SD = 31.50) in time from the actual vanishing time of kick motion. Both the mean magnitudes of RM estimated at kick-typeJT (in frame) were significantly larger than zero (*t*_11_ = 5.874, *p* < 0.001, Cohen’s *d* = 1.696 for the club member group; and *t*_11_ = 2.793, *p* = 0.009, Cohen’s *d* = 0.806 for the non-member group), whereas the group difference in the mean magnitudes of RM estimated at kick-typeJT was not significant (*t*_22_ = 1.052, *p* = 0.304, Cohen’s *d* = 0.430). Furthermore, as seen in Figure 7C, the magnitude of RM in the non-member group calculated at the club member’s kick-typeJT (−27.7 frame position) was 3.9 frames (SD = 3.58), which was not significantly different from that (4.4 frames) of the club member group (*t*_22_ = 0.403, *p* = 0.691, Cohen’s *d* = 0.164). The magnitude of RM in the club member group at the non-member’s kick-typeJT was 3.6 frames (SD = 2.22), which was not significantly different from that (3.1 frames) in the club member group (*t*_22_ = 0.441, *p* = 0.663, Cohen’s *d* = 0.180).

To examine the perceived (or judged) frame positions at which kick-typeJT arose, we calculated them for each participant by adding the individual kick-typeJT and magnitude of the RM estimated at kick-typeJT. Subsequently, the mean perceived (or judged) frame positions at which kick-typeJT arose were calculated for the club member and non-member groups (Figure 8B). The results were −23.3 (SD = 2.51), namely −27.72 (mean kick-typeJT) plus 4.44 (mean magnitude of RM estimated at kick-typeJT), for the club member group and −19.2 (SD = 3.50), namely −22.22 (mean kick-typeJT) plus 3.05 (mean magnitude of RM estimated at kick-typeJT), for the non-member group. The group means of the perceived (or judged) frame position at which kick-typeJT arose differed significantly between the club member and non-member groups (*t*_22_ = 3.295, *p* = 0.003, Cohen’s *d* = 1.345). Typical kick-motion pictures corresponding to the mean perceived (or judged) frame positions at which kick-typeJT arose for the club member and non-member groups are shown in Figure 8C.

### Correlations between kick-type judgment threshold, coincidence timing error and representational momentum magnitude (estimated at kick-type judgment threshold)

**Figure 9:**
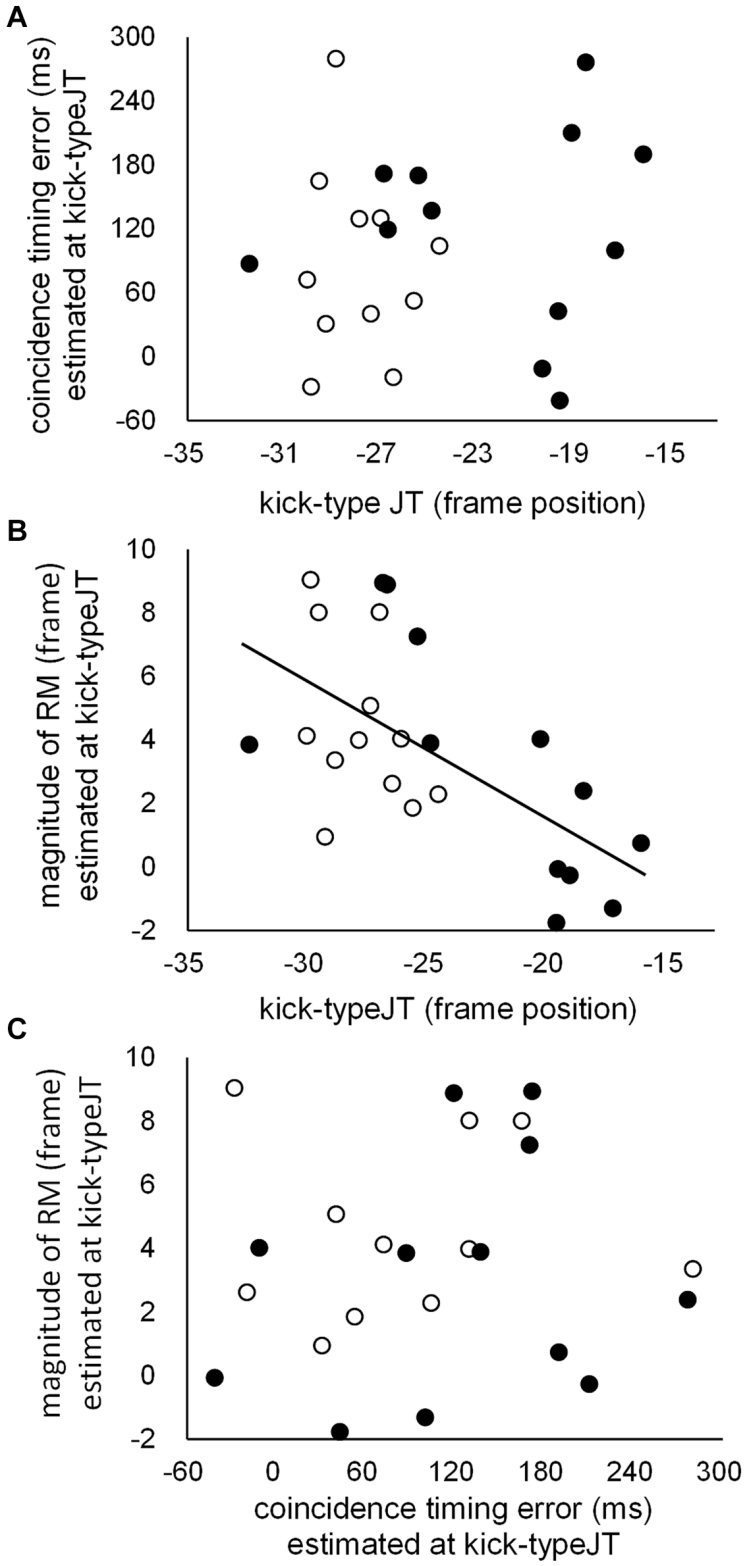
Scattergrams for individual task scores between **(A)** kick-typeJTs and CT errors estimated at kick-typeJT, **(B)** kick-typeJTs and the magnitudes of RM estimated at kick-typeJT, and **(C)** CT errors and the magnitudes of RM estimated at kick-typeJT for club members (unfilled dots) and non-members (filled dots). The regression line depicted in **(B)** indicates that the correlation coefficient is significant.

Correlation coefficients were calculated between kick-typeJT, CT error estimated at kick-typeJT, and the magnitude of RM estimated at kick-typeJT based on all data from the club member and non-member groups. The correlation analyses showed a significant correlation between kick-typeJT and the magnitude of RM estimated at kick-typeJT (Figure 9B, *R* = −0.617, *p* = 0.001). No other significant correlation was found between kick-typeJT and the CT error estimated at kick-typeJT (Figure 9A, *R* = 0.137, *p* = 0.524), or between the CT error estimated at kick-typeJT and magnitude of RM estimated at kick-typeJT (Figure 9C, *R =* 0.075, *p* = 0.728).

We further examined the correlation coefficients between kick-typeJT and the magnitude of RM estimated at kick-typeJT for club member and non-member groups. We found correlation coefficients of −0.408 (*p* = 0.094) and − 0.707 (*p* = 0.005) for the club member and non-member groups, respectively. In addition, the correlation coefficient between the CT error estimated at kick-typeJT and perceived (or judged) frame position at which kick-typeJT arose was not significant (*r* = 0.240, *p* = 0.259).

## Discussion

In this section, we first discuss the results of the overall features of CT error, kick-typeJT, and the magnitude of RM, and then the respective relationships between these three variables. We further describe the overall issue of action anticipation and RM with respect to some correlates of motor simulations. Finally, we explore methodological issues and the limitations of this study.

## Overall features of coincidence timing error, kick-type judgment, and representational momentum magnitude in the club member and non-member groups

The two-way repeated-measures MANOVA on the three dependent variables (Figure 2) showed significant main effects for the group (club member and non-member groups) and vanishing frame position (10 frame positions). The significant group effect indicates that the overall features of the club member and non-member groups generally differ. However, subsequent separate ANOVAs on the three dependent variables showed that the group effect was only significant for kick-type judgments, with no significant group difference emerging for either the CT error or magnitude of RM. For the significant effects of vanishing frame position, the respective task performances (CT error, kick-type judgment, and the magnitude of RM) significantly varied among the vanishing frame positions in different ways for the three tasks.

### Coincidence timing error

The overall mean CT error (98.2 ms, which was significantly larger than zero, see Figure 2A) indicates a significant delay beyond the actual arrival of kick impact. This was also the case for the mean CT errors, 43.6 ms (SD = 69.0) at frame 0 (i.e., kick impact), which is comparable with the CT errors (approximately 20–50 ms delays) reported in Nakamoto et al. (2015).

For group differences, we initially expected that CT errors might be smaller (i.e., more accurate) in the club member group than in the non-member group. However, this was not statistically evident when analyzing either the overall CT errors among the 10 vanishing frame positions (Figure 2A) or CT errors estimated at kick-typeJT (Figures 5C, 6). The literature on CT in various interceptive sports such as tennis and baseball showed that CT errors with the arrival of a moving stimulus at a destination point such as the points where hitting and catching a thrown ball occur were smaller (by approximately half) for skilled players than for novices (Ripoll and Latiri, 1997; Benguigui and Ripoll, 1998; Le Runigo et al., 2005; Lobjois et al., 2005; Nakamoto and Mori, 2012; Nakamoto et al., 2013, 2015). Our results on CT errors at kick-typeJT with the arrival of the kick impact were not statistically consistent with previous findings on CT errors in interceptive tasks such as in tennis and baseball. The reason for the inconsistent results between our study and previous findings for group differences in CT errors is unclear. However, this may be because the nature of the trajectories of the experimental stimuli (i.e., kick motion and the thrown ball) could differ. The trajectory of a kick motion is limited by the length of the leg and other biomechanics, whereas the trajectory of a thrown ball can continue until an external barrier is reached or its kinetic energy is dissipated. Such largely different trajectories of moving stimuli used in the experiments might lead to different results for CT errors, although this should be further examined in future studies.

No significant group difference in CT errors (both overall CT errors and those estimated at kick-typeJT) was found in the present study. Although the reason for this is unclear, it might be because the effective defending action against an opponent’s kick in actual taekwondo may not be the type of interceptive action at the kick impact point (i.e., at the point where the kick is assumed to hit the defender’s body); rather, taekwondo players successfully avoid a possible hit by the opponent’s kick as early as possible prior to its impact point. Therefore, the CT task with the arrival of the kick impact used in our experiment may not replicate the actual situation in a taekwondo defending action. Thus, this may not have been significant for group differences in CT errors for the club member and non-member groups. Defensive actions against opponent’s kicks in taekwondo should likely be performed far before their impact point. Much earlier and more accurate anticipatory judgments for the kick type (and its forthcoming trajectory) may be essential for successful defense, which is consistent with our results of the significantly earlier kick-typeJTs and more accurate judgments for kick types evident in the club member group.

### Kick-type judgment

The rate of correct kick-type judgments increased from around 50% (16 out of 32) at the −54 frame position to almost 100% (near 32) at frame 0 (see Figure 2B). This indicated that the correct kick-type judgments may well be fitted to the theoretical psychometric function curve. Thus, the individual judgment thresholds were estimated at the 75% point, which was defined in this study as the kick-typeJT (see Figures 3, 4). In the individual psychometric function curves, a large difference in variance appeared for the club member (Figure 3A) and non-member (Figure 3B) groups. The reason for this large variance in individual psychometric function curves for the non-member group could be that their sports experiences were incongruent with the experimental stimuli (i.e., taekwondo kicks). Recall that their sports experience was wide ranging, including in tennis, table tennis, baseball, football, and track and field. In contrast, club members’ sports experiences, namely taekwondo *per se*, were congruent with the experimental stimuli of taekwondo kicks. This may have resulted in the large difference in variance between club members and non-members regarding individual psychometric function curves.

Based on the significant group difference in kick-type judgments, the club member group judged kick types more accurately than the non-member group. Furthermore, the significant group difference in kick-typeJT indicated that the club member group successfully judged kick types earlier than the non-member group. Furthermore, the correct judgment rates for kick types were significantly higher in the club member group than in the non-member group for each respective kick-typeJT.

Our results of superior kick-type judgments and kick-typeJT in the club member group support previous findings on combat sports, namely that skilled players can anticipate future outcomes earlier than less-skilled players (Williams and Elliott, 1999; Mori et al., 2002; Hagemann et al., 2010; Rosalie and Müller, 2013; Shin and Lin, 2016). Specifically, previous findings on karate athletes’ anticipation (Mori et al., 2002) showed that choice reaction times in response to either the opponent’s upper or lower attack (presented with a video clip) were significantly shorter for karate athletes than novices, whereas this was not the case for choice reaction times tested in response to the stimulus of a circular figure appearing at either the upper or lower location. This suggests that the early anticipatory judgments of karate athletes may result from domain-specific expertise, rather than from general abilities. Our results of significantly earlier and more accurate spatial anticipatory judgments for opponents’ kick types in the club member group may be underpinned by their domain-specific experiences of taekwondo rather than the general anticipatory ability developed through various other types of sports experiences. This is because the participants of the club member and non-member groups did not differ in the number of years spent in various sports activities. The only difference was that club members had taekwondo experience.

For domain specificity, the postural information picked up by club member participants while observing the kick motion may be effective. Skilled athletes tend to use postural information as a cue to infer future outcomes (Smeeton et al., 2019, for a review) such as kick directions in soccer (Savelsbergh et al., 2005), service directions in tennis (Abernethy and Zawi, 2007), and the type of opponent’s attack in karate (Milazzo et al., 2016). Similar to these findings, the participants in the present study may also have used postural information regarding high and middle kicks (see Figure 1). Participants likely used postural cues as spatial information inherent in the opponent’s unfolding kick action to spatially anticipate kick types at an early phase of the kick motion presented. They may furthermore have used the postural information they perceived earlier in the kick motion more effectively than non-member participants, although the kick-motion postures defined at the kick-typeJT differed only subtly (see Figures 4B, 8C). This indicated a type of domain specificity that resulted in significantly earlier kick-typeJT and more accurate kick-type judgments in the club member group than in the non-member group.

### Representational momentum magnitude

The magnitude of RM (Figure 2C) gradually decreased toward the frame 0 (kick impact) vanishing position. The overall mean magnitude of RM (4.12 frames) was significantly larger than zero, and the mean magnitude (−0.98 frames) of RM at frame 0 (impact point) was also significantly different from zero. An explanation of the small magnitudes of RM found at frame 0 is that RM decreases if participants expect a target to stop (e.g., Finke et al., 1986). Given that our participants would have expected the kick motion to stop at frame 0, the magnitude of RM was reduced for those frame positions closer to frame 0. Furthermore, in the adjustment method used in this task, participants chose either the forward or backward frame image until their last decision. Since the kick impact frame was operationally defined as the frame in which the kick (foot) swinging motion switched from the forward to backward phase, any choices of forward and backward frames around kick impact resulted in similar kick posture images. This may be the reason for the small magnitudes of RM appearing at the 0 (and − 8) frame positions.

The mean magnitudes of RM estimated at kick-typeJT (Figure 8A) were also significantly larger than zero for both the club member and non-member groups. This indicates that both groups perceived vanishing frame positions with significant forward displacements in the direction of motion; thus, the RM was significantly occurring. The mean magnitudes of RM estimated at kick-typeJT in the club member and non-member groups were 4.4 and 3.1 frames, respectively, corresponding to 36.7 and 25.8 ms, respectively, after the vanishing time of kick motion. This temporal range of the magnitude of RM seems much smaller than those (185.0 and 67.5 ms) for basketball experts and novices (Gorman et al., 2012). In contrast, it is relatively similar to the magnitudes of RM of 80 ms and 30 ms reported by Nakamoto et al. (2015) for baseball players and novices, respectively. We speculate that a possible reason for such large ranges of the magnitude of RM in different studies might be the different durations of the stimulus presentation. Gorman et al. (2012) presented video clips of 7 s duration containing 10 basketball players in a game on a basketball court as their experimental visual stimuli, while Nakamoto et al. (2015) used an apparent linear movement of a visual stimulus (like a thrown ball) traversing 4 m in a very short duration of 266 or 400 ms. In the present study, the mean duration of the high and middle kicks was 339.5 ms from the toe-off of the kicking foot from the floor to the assumed kick impact. This range of stimulus presentation duration is more similar to that of Nakamoto et al. (2015) than that of Gorman et al. (2012). This short duration of visual stimuli might be a reason for the resultant small (short duration) magnitudes of RM found in Nakamoto et al. (2015) and in the present study, although this should be examined in future studies. The most important point of our results regarding the magnitudes of RM was that both groups showed significantly larger magnitudes (greater than zero) of RM, indicating that they perceived the vanishing frame positions of the kick motion as being shifted forward in the direction of motion.

We failed to find significant group differences in the magnitude of RM between the club member and non-member groups. This is inconsistent with the findings of previous studies on RM among experts such as athletes in baseball (Nakamoto et al., 2015), basketball (Gorman et al., 2011, 2012), badminton (Jin et al., 2017), rugby (Anderson et al., 2019), dance (Chen et al., 2019), aircraft pilots (Blättler et al., 2011), and automobile drivers (Blättler et al., 2010, 2012), all of which showed significant group differences between experts and novices.

There are at least two possible reasons for the absence of a significant group difference in the magnitude of the RM in the present study. First, we speculate that since our participants in the non-member group were not novices of sports or non-athletes, the mean length of general sports experience (including taekwondo experience) did not significantly differ between the club member and non-member groups. Furthermore, the sports experiences of the non-members included many ball sports such as tennis, table tennis, baseball, and football, with track and field being the only exception, which only two participants in the non-member group had experienced. In general, players of ball sports frequently observe fast-moving objects in their games, which would develop their ability to track these objects. Reportedly, RM typically occurs while visually tracking a moving stimulus (although RM also occurs with implied motion and with frozen-action photographs, where eyes are fixated on a given stimulus; Kerzel, 2000; Hubbard, 2005). Therefore, the non-members’ experiences of ball sports may have developed some perceptual ability to visually track a moving stimulus, even though it is not domain-specific to ball sports. This could lead to producing significant RM, even when measuring RM with non-domain-specific stimuli (i.e., with kick motion stimuli). Consequently, the potentially larger magnitude of RM among club members would be offset, and no significant group differences would be found in this regard. Furthermore, as shown in the Appendix, nine non-members and three club members reported that when watching the kick motion stimuli, the body part they focused on was the “foot.” However, our examination showed no significant effects regarding the behavior of focusing on the “foot” on the magnitude of RM (or on CT errors). Therefore, this gaze behavior may not have affected the magnitude of RM, and is thus not a causal reason for the fact that no group difference in RM was found. Further exploration in a future study is needed in this regard.

A second possible reason for the absence of significant group differences in the magnitude of RM can be explained in terms of the magnitude of RM estimated at kick-typeJT not being due to the effects of experts’ top-down information (rather, it might be due to bottom-up perceptual information of the observed motion). The top-down information includes expectations and prior knowledge of observed motion, which would contribute to underpinning the development of RM. Hudson et al. (2016a) and Hudson et al. (2016b) reported that the magnitude of RM while observing the reaching motion of another person was greater when the actual motion was congruent with the motion expectation induced by the action goal than when it was incongruent, even when observing identical motions in both congruent and incongruent conditions (c.f., Anderson et al., 2019, for sports situations). In contrast, skilled combat players are better at predicting the features of an opponent’s attack using prior knowledge such as the situational probability of the occurrence of a certain attack event even before an actual attack occurs (Rosalie and Müller, 2013; Milazzo et al., 2016). These findings suggest that the larger magnitude of RM arising among sports experts (with domain-specific stimuli) may, in part, be due to experts’ higher degree of top-down information on motion expectations or prior knowledge of events specific to their sports situations. However, this was not observed in the present study. In our examination of spatial anticipation for kick type judgments, we analyzed the kick-typeJT, which was set at the same 75% correct judgment rate for the club member and non-member groups. This resulted in no bias due to the degree of prediction or expectation for upcoming kick types. Furthermore, the kick types were randomly presented in this study; thus, no bias occurred in the situational probability of presenting the high and middle kicks. Therefore, neither top-down motion expectation nor prior knowledge of situational probability influenced the magnitude of RM in this study, resulting in no significant group differences in the magnitude of RM.

### Significant relationship between kick-type judgment threshold and representational momentum magnitude (estimated at kick-type judgment threshold)

In this section, likely implications in the significant relationship (*r* = 0.617, *p* = 0.001) between kick-typeJT and the magnitude of RM estimated at kick-typeJT (Figure 9B) are highlighted. For the significant relationship between the magnitude of RM and judgment threshold, we speculate that a common factor may enhance both kick-typeJT and the magnitude of RM estimated at kick-typeJT. The original theory of RM (Freyd and Finke, 1984; Hubbard, 2005, for a review) hypothesizes that the representation of a moving object in our memory is dynamic rather than static, such that it continues to move forward in the direction of its motion, similar to the dynamic environmental feature of the moving object. Accordingly, the hypothesized nature of the forward-shifting memory representation of the kick motion could be beneficial in anticipating kick types at an early phase of the kick motion and in yielding a large magnitude of RM, which may be a common factor causing the significant relationship between kick-typeJT and the magnitude of RM estimated at kick-typeJT.

From another perspective, an RM-based anticipation mechanism likely contributes to better action anticipation, which is developed to compensate for neural delays in the perceptual and visual processes (Kerzel and Gegenfurtner, 2003; Nijhawan and Kirschfeld, 2003; Hubbard, 2005) of moving objects or events including human actions. In other words, anticipation mechanisms are essential for better human perception and actions performed during processes with neural delays, and RM-based anticipation may contribute to these processes. For RM-based anticipation, Verfaillie and Daems (2002) examined the priming effects of the observation of human-motion animations on reaction times (RTs) in judging the features (whether they were possible or impossible postures performed as a dummy task irrelevant to the examination of priming effects) of test stimuli for certain body postures. The test stimuli for human body postures were either the end-phase postures of human-motion animations with slightly forward-shifted displacements (future-posture prime conditions) or the starting-phase postures of human-motion animations with slightly backward-shifted displacements (past-posture prime conditions). Their results showed that much shorter RTs appeared when judging the test body postures of the ending-phase postures with forward-shifted displacements than when judging those of the starting-phase postures with backward-shifted displacements. This suggests that the observation of human-motion animations may have yielded typical RM and thus facilitated anticipation of the ending-phase body postures with forward displacements (rather than the starting-phase body postures). Accordingly, based on our results, we speculate that a future scene of the ongoing kick motion may have been perceived using RM, thus anticipating the kick type of the ongoing kick motion and resulting in a significant relationship between kick-typeJT and the magnitude of RM estimated at kick-typeJT.

Interestingly, although the overall correlation (for all participants from both groups) between kick-typeJT and the magnitude of RM estimated at kick-typeJT was significant, this effect was driven by the non-member group (*r* = −0.707, *p* = 0.005; for the club member group, *r* = −0.408, *p* = 0.094). It is likely that for the non-member group, a common factor such as the hypothesized nature of the forward-shifting memory representation of kick motion might have enhanced both the spatial anticipatory judgment of kick types (i.e., kick-typeJTs to be earlier) and magnitude of RM (to be larger). In contrast, for the club member group, the influence of the common factor on spatial anticipation might be relatively weak. The excellent spatial anticipation of club members might depend less on RM and more on other factors such as postural information as a domain specificity in club members (as discussed in the previous section). This may be a reason for the modest, non-significant correlation coefficient in the club member group; however, this should be examined in future studies.

### Insignificant relationships between coincidence timing error and representational momentum magnitude (estimated at the kick-type judgment threshold)

The insignificant relationship between the CT error estimated at kick-typeJT and magnitude of RM estimated at kick-typeJT (Figure 9C) indicates that no essential common factors exist between them. This suggests that the accuracy of CT with the arrival of the kick impact was not facilitated, even when participants showed significant magnitudes of RM, in other words, even when they perceived the vanishing frame position of the kick motion at a forward-shifted position (closer to the kick impact point as the target in the CT task). This result is inconsistent with previous findings for baseball situations, in which CT errors with the arrival of a visual stimulus at a destination point were significantly correlated with the magnitudes of RM (Nakamoto et al., 2015). A likely reason for the inconsistent findings regarding the relationship between CT error and RM might be the characteristics of the different trajectories of the kick motion and thrown ball stimuli used in the respective experiments, as already discussed elsewhere.

Alternatively, another reason can be speculated in terms of the different implications of the CT tasks for the taekwondo and baseball situations in both the present study and Nakamoto et al. (2015). In baseball, temporal anticipatory judgments (CT errors) of a moving stimulus may be essential in successfully performing an interceptive action such as hitting and catching a fast-moving ball arriving at its destination point (e.g., Kidokoro et al., 2019). Therefore, the large magnitude of RM (i.e., the large forward displacements in anticipation of the future position of the moving stimulus) could help enhance CT accuracy (or decrease CT errors) at the destination point. In contrast, in taekwondo, CT errors were examined for the arrival of the kick impact at the destination point, and such a CT action (i.e., an assumed interceptive action at the arrival of the kick impact) may not be crucial for a successful defense against the opponent’s attack. Rather, much more quick judgments of kick types in an ongoing opponent’s attack (and thus kick trajectory) must significantly contribute to performing a successful defense. Mori et al. (2002) reported that karate athletes discriminated opponents’ upper and lower attacks significantly earlier than novices. Therefore, it is likely that the magnitude of RM *per se* may not have contributed to enhancing the accuracy of the CT performance with the kick impact. This is a possible reason for the different findings between baseball and taekwondo regarding the relationship between the magnitude of RM and CT error.

Given that CT errors differ for baseball and taekwondo, and that early judgment of an opponent’s attack is essential in defense action in taekwondo, the potential functional relationships between the magnitude of RM and CT errors may be domain-specific. Nakamoto et al. (2015) provided empirical evidence that a larger RM in baseball players than in novices appeared only for linear movements of stimuli, but not for rotary ones. Here, the nature of the linear movements of stimuli is domain-specific to baseball, but rotary movements may not be. Therefore, the relationship between the magnitude of RM and CT error in sports situations may differ for different sports and thus vary according to domain-specific rather than general characteristics.

### Insignificant relationships between the kick-type judgment threshold and coincidence timing error (estimated at the kick-type judgment threshold)

Regarding the relationship between kick-typeJT and CT error estimated at kick-typeJT (Figure 9A), we provisionally predicted that accurate CT performance (i.e., smaller CT errors) might result from earlier kick-typeJT (i.e., earlier spatial anticipation of the kick type). Nevertheless, the correlation analysis did not support our provisional prediction, indicating that CT errors estimated at kick-typeJT were not associated with the early or late appearance of kick-typeJT. This was also replicated in the resultant insignificant relationship between the CT error and perceived or judged frame position at which kick-typeJT arose (i.e., kick-typeJT + the magnitude of RM estimated at kick-typeJT). The lack of a relationship between spatial and temporal anticipatory judgments suggests that the accuracy of temporal anticipation of kick impact may not result from early or late spatial anticipation of the kick type. This is because temporal and spatial anticipatory judgments may differ in their functional roles in defending an action against the opponent’s attack in taekwondo: the temporal judgments with the arrival of kick impact (CT) may not be beneficial (and may be too late) in successfully defending action in taekwondo, whereas the spatial judgments of kick types (kick-typeJT) may be critical in a successful defense in avoiding an opponent’s attack before its arrival (i.e., to hit the defender’s body), as discussed in the previous section. This is a possible reason for the non-significant relationship between kick-typeJT and CT errors estimated at kick-typeJT found in this study.

Furthermore, a possible reason for the lack of a relationship between kick-typeJT and CT error estimated at kick-typeJT is that different sources of information such as postural and motion information may be used differently for spatial and temporal anticipatory judgments. Skilled athletes generally tend to use postural information (e.g., the orientation of the opponent’s posture) as anticipatory cues to infer future outcomes (Smeeton et al., 2019, for a review) such as those in soccer (Savelsbergh et al., 2005), tennis (Abernethy and Zawi, 2007), and karate (e.g., Milazzo et al., 2016). This was also the case in the present study, providing evidence that the club member group successfully judged kick types at an earlier phase of the kick motion than the non-member group, even though there was only a subtle group difference in the kick-motion postures used in the successful kick-type judgments (see Figures 4B, 8C). In contrast, motion information can be used to accurately estimate the arrival time of dynamic events such as TTC. The literature on TTC suggests that multiple sources of spatial and temporal information such as tau (Lee, 1976; Bootsma and van Wieringen, 1990), retinal disparity (Rushton and Wann, 1999; van der Kamp et al., 1999), the ratio between perceived distance and velocity (e.g., Smeets et al., 1996), and internal models (e.g., Zago et al., 2004) can be effectively used to accurately estimate TTC, a type of temporal anticipation.

Regarding the use of spatial and temporal information for accurate motion perception, Lange and Lappe (2007) suggested that although both spatial and temporal information would generally underpin the accurate perception of biological motion, spatial information was more essential than temporal information in detecting the left and right directions of a walker. In contrast, spatial and temporal information contributed to successful discrimination in detecting the forward and backward directions of walking. Lappe (2012) further suggested that for the perception of biological motion, visual inputs were first used to estimate the form and posture of the human body, as shown by biological motion. Then the motion was estimated by detecting the sequential change of body form and posture with a motion vector. These findings suggest that postural and motion information can be used differently for the spatial and temporal anticipatory judgment of human motion. Accordingly, postural and motion information may have been used differently to anticipate the spatial (kick types) and temporal (CT errors) features of the kick motion, thus resulting in no significant relationships between kick-typeJT and CT errors estimated at kick-typeJT. Details of postural and motion information use in spatial and temporal anticipatory judgments of the kick motion should be further examined in future studies.

### Action anticipation and representational momentum: likely correlates of motor simulation

In this section, *action anticipation*, which is characterized by both the spatial and temporal aspects of anticipation, is discussed in terms of RM and *motor simulation*. Motor simulation is thought to play an important role in enhancing skilled athletes’ efficient reading of observed action kinematics. It involves spatial and temporal aspects, thereby improving action anticipation (Aglioti et al., 2008; Cañal-Bruland et al., 2012; Urgesi et al., 2012; Mulligan and Hodges, 2014; Makris and Urgesi, 2015; Mulligan et al., 2016a,b; Nakamoto et al., 2021). Motor simulation may occur during action observation in the mirror neuron system, which is activated by the actual execution of an action similar to the observed action (Decety et al., 1994; Gallese et al., 1996; Blakemore and Decety, 2001; Gallese, 2005; Urgesi et al., 2010; Gallese and Sinigaglia, 2011), thereby matching action observation and execution (di Pellegrino et al., 1992; Rizzolatti and Craighero, 2004). The ability to read the observed action kinematics and action anticipation are developed through long-term motor experiences of actions similar to the observed action (Diersch et al., 2012; Mulligan and Hodges, 2014; Mulligan et al., 2016a,b). This means that both the motor experience of actions and observation of motor actions may contribute to developing motor simulation ability. Therefore, skilled athletes can successfully read others’ action kinematics in motor simulation, which improves their action anticipation.

Furthermore, motor simulation can be interpreted in terms of the nature of the implied motion, which induces RM by viewing a static image of human actions, similar to RM induced by viewing actual human actions or moving objects. Implied motion is thought to activate the motor system through the mirror neuron system, yielding dynamic information from static images of human actions in terms of motor simulation (Urgesi et al., 2006, 2010). Therefore, motor simulation processes may be essential in facilitating both RM and action anticipation when observing human actions. Our results on the significant relationship between kick-typeJT and RM provide evidence for the essential role of RM as an influential factor in action anticipation, which is underpinned by motor simulation processes. This view is consistent with recent findings on the *predictive* coding of human actions (in event coding theory) in explaining action anticipation concerning RM (Anderson et al., 2019). However, these accounts should be further examined in future studies.

### Methodological issues

#### Estimation of individual kick-type judgment thresholds

In this study, individual kick-typeJT was determined as the frame position where the correct judgment rate for kick types was 75%, the midpoint between chance (50%) and perfect level (100%), in terms of individual psychometric function curves. This enabled us to theoretically estimate the threshold (i.e., kick-typeJT) for the anticipatory judgment of kick types at the 75% correct judgment rate. In contrast, in the traditional method (e.g., Farrow et al., 2005), the threshold of correct judgment was identified at a “predictable time point,” where a significant improvement in anticipation accuracy would occur across two successive vanishing points. In this traditional case, the time windows of a certain time interval were arbitrarily preset by the experimenter as experimental conditions to examine anticipation or prediction accuracy. An individual threshold of anticipatory judgment was then identified at one of the preset time windows for participants’ successful judgments. Therefore, the predictable time point for the anticipation of a target event would be estimated as a relatively wide-ranging anticipation time window. Within the relatively large time window used in traditional methods, it would be difficult to detect individual differences in predictable time points as an anticipation capability. The literature (Rosalie and Müller, 2013) suggests that even though individual differences in expertise (e.g., predictable time point) are quite small, the successful detection thereof is vital in uncovering the underlying mechanisms of anticipation. In this study, the significant group difference in kick-typeJT between the club member and non-member groups seemed to form only a subtle difference in kick-motion postures (Figure 4B), nevertheless contributing to the significant differences in kick-typeJT. Therefore, it is likely that using psychometric function curves to determine individual kick-typeJT is a worthwhile method for examining anticipation capability, especially in time-constrained sports (Gray, 2010).

#### Estimation of individual coincidence timing error and representational momentum magnitude estimated at the kick-type judgment threshold

Similar to the use of psychometric function curves to estimate individual kick-typeJTs, we used a similar methodology to estimate the CT error and magnitude of RM estimated at kick-typeJT, with the second-degree equation function curves (Figures 5, 7) fitted to the original task scores (CT errors in response time and displacements in frames for vanishing frame positions). However, there were no theoretical equation functions for the CT errors and magnitudes of RM. The results of the original task scores showed that both the CT error scores (Figure 2A) and magnitudes of RM (Figure 2C) at 10 vanishing frame positions tended to vary in the shape of second-degree equation function curves; therefore, we calculated individual second-degree equation function curves for the CT error (Figure 5) and magnitude of RM (Figure 7). Based on these curves, we optimally determined the individual CT error and magnitude of RM estimated at kick-typeJT per participant, with no immediate influence from actual variations in the original or raw task scores. The suitable methods for some second- and third-degree equation function curves may be worthwhile in determining estimations at specific points of task conditions with less influence owing to existing variations in raw task scores.

### Limitations of the present study

Although this study provides several new findings on the relationship between the magnitude of RM and spatial or temporal anticipatory judgments in taekwondo, a methodological limitation resulted from the trial orders for the three experimental tasks. Three tasks, namely CT with the arrival of the kick impact, judgments of kick types, and judgments of vanishing frame position (which was used to estimate the magnitude of RM) were sequentially performed with different delays from the vanishing time of the kick motion video clips as visual stimuli. The CT task was performed immediately after the kick motion vanished, and the kick-type judgment task was subsequently performed, followed by the judgment of the vanishing frame position. The last two tasks were performed based on participants’ short-term memory of the last image of the presented kick video clip, although the delays (i.e., retention intervals) from the vanishing time of the kick motion to the execution of the tasks were relatively short. Kick-type judgments may not be affected by delays, as participants could easily remember the kick images. However, there may be some problems in measuring the magnitude of RM. It has been suggested that the magnitude of RM decreases with relatively long retention intervals such as 500–900 ms (Freyd and Johnson, 1987). This implies that in the present study, the magnitude of RM may have been affected by the elapsed time from the stimulus vanishing to the measurement of RM, as the measurement of RM was performed as the last task. The relatively long retention intervals from stimulus vanishing to the measurement of RM may have resulted in the decreasing RM magnitudes. In addition to the long retention interval, the adjustment method used in estimating the vanishing frame position also took a few seconds. This may further have decreased the magnitude of RM. In this regard, the fixed order of tasks (thus resulting in relatively long retention intervals in measurements of RM) may be a problematic limitation in the present study. Nevertheless, the resultant magnitudes of RM in this study were significantly larger than zero in both the club member and non-member group, indicating that RM significantly occurred under such conditions despite relatively long retention intervals.

To avoid the limitation derived from the fixed order of tasks in future studies, each task should be performed immediately after observing a kick video clip as a visual stimulus, such that the presentation of a kick video clip is followed by one of the three tasks in separate experimental procedures. Alternatively, the measurement of RM should be immediately performed after either the CT task or kick-type judgment task in separate experimental procedures to examine the possible effects of RM on the CT or kick-type judgment task without relatively long retention intervals. This would result in larger magnitudes of RM and perhaps in a significant correlation between the CT errors and magnitudes of RM, which was not evident in this study. Thus, these experimental procedures serve as suggestions for future study.

## Data availability statement

The original contributions presented in the study are included in the article/Supplementary material, further inquiries can be directed to the corresponding author. The video clips used in this study are available from Figshare at: http://doi.org/10.6084/m9.figshare.23542824.

## Ethics statement

The studies involving humans were approved by The Ethics Committee of the Tokyo Metropolitan University. The studies were conducted in accordance with the local legislation and institutional requirements. The participants provided their written informed consent to participate in this study. Written informed consent was obtained from the individual(s) for the publication of any potentially identifiable images or data included in this article.

## Author contributions

The experimental design and software program were provided by KI. TS conducted the experiments. The first draft of the manuscript was written by KI. The Introduction and Discussion sections on the issues of anticipation were primarily written by HN. All authors contributed to the article and approved the submitted version.

## Funding

This study was supported by a Grant-in-Aid for Scientific Research from the JSPS (grant numbers 17H00875 and 21K18562) to KI.

## Conflict of interest

The authors declare that the research was conducted in the absence of any commercial or financial relationships that could be construed as a potential conflict of interest.

## Publisher’s note

All claims expressed in this article are solely those of the authors and do not necessarily represent those of their affiliated organizations, or those of the publisher, the editors and the reviewers. Any product that may be evaluated in this article, or claim that may be made by its manufacturer, is not guaranteed or endorsed by the publisher.

## Footnotes

^1^The required sample size was calculated using G^*^Power, version 3.1.9.2, for the F tests - ANOVA (repeated measures, within-between interaction). The predetermined input parameters were: effect size *η_p_*^2^ = 0.58 (*f* = 1.1751393), from the effect size for the group effect of baseball players and novices on the magnitude of RM reported by Nakamoto et al. (2015); *α* = 0.05; power (1 – *β*) = 0.95; the number of groups = 2 (club member and non-member groups); number of measurements = 10 (measurements at 10 vanishing frame positions); and non-sphericity correction ε = 1/(10–1) = 0.11112, as a lowest ε, which was assumed in terms of repeated measurements at 10 vanishing frame positions. This resulted in a total sample size of 12.

^2^The mean numbers of frames from the toe-off to the kick impact for the high and middle kicks were 41.9 ± 2.91 and 39.6 ± 2.94 frames, respectively, which significantly differed between each other (*t_79_* = 7.89, *p* < 0.01, Cohen’s *d* = 0.882). In this study, 160 video clips of high and middle kicks (80 video clips each) were randomly presented for two consecutive sessions for a total of 320 video clips, after which participants performed 3 experimental tasks. Analyses were conducted for all dependent variables based on the collapsed data across both video clips of high and middle kicks.

^3^The resultant numbers of canceled and restarted CT trials were 19.3 (SD = 33.2) and 12.2 (SD = 21.4) on average for the club member and non-member groups, respectively, which did not significantly differ from each other (*t*_22_ = 0.629, *p* = 0.536, Cohen’s *d* = 0.257). For club members, one participant canceled and restarted 112 CT trials of the 320 total CT trials, and the actions of others in this regard ranged from 0 to 48 trials. For non-members, one participant canceled and restarted 76 CT trials of the 320 in total, and the actions of others in this regard ranged from 0 to 23 trials. Therefore, it was evident that only two participants canceled and restarted a large number of CT trials, whereas most participants did only a small number of CT trials. This resulted in no significant difference in the number of canceled and restarted CT trials between the club member and non-member groups.

^4^The reason for the fixed rather than counterbalanced order of the three tasks was that for the CT task, participants were asked to press the response button on their CT with the arrival of the assumed kick impact; therefore, the CT task was performed first. In estimating the vanishing frame positions (the last task), participants were allowed to select (and thereby watch) subsequent frames beyond the actual vanishing frame according to the adjustment method, where the participants altered (by pressing pre-specified keys on the gamepad) the frame position presented as a probe frame into either the previous or subsequent frames according to their estimation of the vanishing frame position. This would have enabled participants to know the type of kick when they moved toward subsequent frames beyond the actual vanishing frame (with no limitation in shifting the frame in either the forward or backward direction in the adjustment method) before performing the anticipatory kick-type judgment task (second task). This is why the anticipatory kick-type judgment task was performed a second time prior to the task of estimating the vanishing frame position.

^5^The results of the examination of the word reported for the body parts provided in the Appendix.
