# Supplementary material for: Relationships between the magnitude of representational momentum and the spatial and temporal anticipatory judgments of opponent’s kicks in taekwondo
Source: Front Psychol. 2023 Sep 21;14:1193116. doi: 10.3389/fpsyg.2023.1193116 (PMC10551154; doi:10.3389/fpsyg.2023.1193116)
Supplement: Supplementary file 1 [file Data_Sheet_1.docx]

**Appendix**

*Subjective reports of body parts that participants focused on while observing the kick motion*

For the questionnaire administered after the completion of the experimental trials, our results showed that participants reported eight words regarding the body parts they focused on while observing the kick motion—the whole body, face, hand, arm, trunk, knee, foot, and leg. The club member and non-member groups reported 1.58 (SD = 0.86) and 2.08 (SD = 0.64) words per participant on average, respectively. Figure 1 shows the number of participants who reported each word for a body part in the club member and non-member groups. A *Chi*-square test performed on these data did not show a significant difference in frequency between the two groups according to body parts (*X*^2^ = 7.954, *df* = 7, *p* = 0.337). Among the eight words for body parts, there was a relatively large variation in the number of participants who reported the word “foot:” three club members and nine non-members (out of 12 participants per group). A similar number of participants in the club member and non-member groups reported words for other body parts.

ANOVAs with two between-participant factors (group, club member and non-member groups; and “foot”: participants who reported the word “foot” and those who did not) were separately performed on the three variables, namely kick-typeJT, CT error at kick-typeJT, and the magnitude of RM at kick-typeJT. The results of the ANOVAs showed no significant main effect of “foot” or any interaction for the three variables. Specifically, for kick-typeJT, the main effect of group was significant (*F*_1, 20_ = 7.667, *p* = 0.012, *partial η*^2^ = 0.277), whereas the main effect of “foot” was not significant (*F*_1, 20_ = 0.297, *p* = 0.596, *partial η*^2^ = 0.014), with no significant interaction between group and “foot” (*F*_1, 20_ = 0.333, *p* = 0.570, *partial η*^2^ = 0.016). For the CT error at kick-typeJT, no significant main effect was found for group (*F*_1, 20_ = 0.463, *p* = 0.504, *partial η*^2^ = 0.023) or “foot” (*F*_1, 20_ = 0.497, *p* = 0.489, *partial η*^2^ = 0.024), with no significant interaction between group and “foot” (*F*_1, 20_ = 0.360, *p* = 0.555, *partial η*^2^ = 0.018). For the magnitude of RM at kick-typeJT, no significant main effect was found for group (*F*_1, 20_ = 0.787, *p* = 0386, *partial η*^2^ = 0.038) or “foot” (*F*_1, 20_ = 0.001, *p* = 0.675, *partial η*^2^ = 0.000), with no significant interaction between group and “foot” (*F*_1, 20_ = 0.118, *p* = 0.735, *partial η*^2^ = 0.006).

**Figure 1.** Number of participants reporting each word of the body part they focused on while observing the video clips.
